# Peri‐implant inflammation increases the risk of osteonecrosis in mice treated with bisphosphonate

**DOI:** 10.1002/JPER.24-0760

**Published:** 2025-04-15

**Authors:** Ana Bujila, Davi N. A. Silva, Sepehr Monajemzadeh, Taciane M. da Silveira, Naseim Elzakra, Maísa Casarin, Kimberly Flores, Clara Magyar, Julie Marchesan, Reuben Kim, Sotirios Tetradis, Flavia Q. Pirih

**Affiliations:** ^1^ Section of Periodontics School of Dentistry University of California Los Angeles California USA; ^2^ School of Dentistry Federal University of Pelotas Pelotas Rio Grande do Sul Brazil; ^3^ Section of Oral and Maxillofacial Radiology University of California Los Angeles California USA; ^4^ Department of Pathology and Laboratory Medicine David Geffen School of Medicine University of California Los Angeles California USA; ^5^ Department of Periodontology School of Dentistry University of North Carolina at Chapel Hill Chapel Hill North Carolina USA; ^6^ School of Dentistry Section of Restorative Dentistry University of California Los Angeles California USA

**Keywords:** alveolar bone losses, bone tissue, dental implant, osteoclast, peri‐implantitis

## Abstract

**Background:**

Bisphosphonates (BPs) are effective in managing bone diseases due to their anti‐resorptive properties but are linked to medication‐related osteonecrosis of the jaw (MRONJ), particularly concerning dental implants. This study explored the combined impact of ligature‐induced peri‐implant inflammation and zoledronic acid (ZA), a BP, using a murine model.

**Methods:**

Twenty‐four mice underwent bilateral maxillary molar extractions and implant placements, with ZA or vehicle treatment and ligature placement on the left side. Two groups were defined: group 1 (vehicle‐treated) with control (Veh‐C) and ligature (Veh‐L) implants, and group 2 (ZA‐treated) with control (ZA‐C) and ligature (ZA‐L) implants. Clinical, micro‐CT, histological, and immunohistochemical analyses were performed. We hypothesized that peri‐implant inflammation elevates MRONJ risk with BP treatment.

**Results:**

Ligature groups showed increased soft tissue edema compared to controls, without differences between vehicle and ZA treatments. The Veh‐L group exhibited significantly greater bone loss than other groups. Histology showed higher inflammatory infiltrate in ligature groups. Osteocyte empty lacunae and osteonecrosis were significantly greater in ZA‐L. Picrosirius red staining revealed disorganized collagen fibers and separation in ZA‐L. Immunohistochemistry showed increased neutrophils (NIMP‐R14+) and monocytes/macrophages (CD11b+) in the ligature groups, with no significant differences between Veh‐C and ZA‐C.

**Conclusion:**

Ligature treatment enhances peri‐implant inflammation, with ZA heightening the risk of MRONJ. These findings highlight the critical importance of early detection and management of peri‐implant inflammation in patients undergoing BP therapy, particularly those at high risk of MRONJ. Clinicians should emphasize preventive measures, such as regular monitoring of peri‐implant health and reducing local inflammatory triggers, to mitigate the adverse effects of BPs on peri‐implant bone health.

**Plain Language Summary:**

Dental implants are a reliable solution to replace missing teeth. However, like natural teeth, implants can develop inflammation around them—peri‐implantitis. Our study found that, when this inflammation occurs in patients taking BPs (a medication commonly used to treat osteoporosis and other bone diseases), the risk of developing a serious jaw condition called osteonecrosis (ONJ) increases significantly. ONJ prevents the jawbone from healing properly, leading to pain, infection, and even exposed bone. These findings highlight the importance of preventing and managing inflammation around dental implants to reduce the risk of complications, especially in patients taking BPs. Our research suggests regular dental check‐ups and proper oral hygiene can help maintain implant health and prevent severe bone‐related conditions. Patients and healthcare providers can take proactive steps to improve long‐term oral health outcomes by understanding these risks.

## 1 INTRODUCTION

Bisphosphonates (BP) are widely acknowledged as effective anti‐resorptive medications for managing osteoporosis and bone neoplasms.^1^ However, according to the American Association of Oral and Maxillofacial Surgeons, BP treatment may lead to medication‐related osteonecrosis of the jaw (MRONJ), a severe complication characterized by exposed bone in the jaw that has persisted for more than 8 weeks.^2^ The prevalence of MRONJ in the United States is estimated to range from 0.1% to 0.2% in individuals receiving oral BPs for over 4 years.^3^ This prevalence varies across different countries, with reported rates of 0.001% in Canada, 0.004% in Scotland, and 0.00038% in Germany.^4^, ^5^

More than half of MRONJ cases have been associated with dental surgical procedures as triggering events.^6^, ^7^ Although the role of dental implant placement or inflammation around implants in the development of MRONJ is less clearly defined, it is generally considered a risk factor.^8^, ^9^ Moreover, the placement of dental implants is contraindicated in patients who have received high doses of BP, particularly those undergoing oncologic treatment^10^.

Given that MRONJ can be triggered by inflammation and infection,^2^ it is plausible that peri‐implant inflammation could exacerbate the risk of MRONJ. Peri‐implantitis is an inflammatory process affecting the soft and hard tissues around dental implants, leading to the progressive loss of supporting bone.^11^ It has been identified as a potential triggering factor for MRONJ, with studies correlating the 2 conditions.^12^, ^13^ Recent research also suggests that peri‐implantitis can increase the risk of MRONJ associated with osseointegrated implants in rats.^14^

Despite evidence suggesting an increased risk of MRONJ in patients with dental implants,^12^, ^15^ research on the impact of inflammatory responses around implants is still limited. Given these considerations, we hypothesize that ligature‐induced peri‐implant inflammation increases the risk of MRONJ when implants are placed before BP administration is initiated. Our study aims to investigate whether ligature‐induced inflammation around dental implant fixtures in mice undergoing oncologic doses of BP treatment increases the risk of MRONJ. This research seeks to clarify the potential interplay between peri‐implantitis and BP treatment in the development of MRONJ, contributing valuable insights to managing patients at risk.

## 2 MATERIALS AND METHODS

### 2.1 Animal care

Twenty‐four 3‐week‐old C57BL/6J male mice* were used according to the ARRIVE guidelines^16^ and a protocol approved by the Chancellor's Animal Research Committee at the University of California, Los Angeles. A sample size of 12 mice per group was determined based on 80% power, a 15% standard deviation (σ), and a 95% confidence interval (α  =  0.05), comparing linear bone loss between the control group (minimal bone loss) and the ligature group (maximal bone loss). The mice were kept under standard conditions with a 12‐h light/dark cycle at 22 ± 0.1°C and had ad libitum access to a soft diet.† A split‐mouth design was applied.

### 2.2 Tooth extraction and implant placement

**FIGURE 1:**
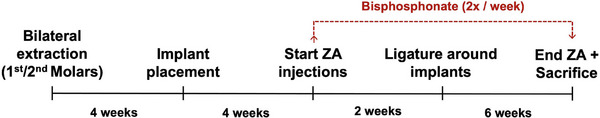
Chronological sequence and experiments performed.

Mice underwent bilateral extraction of their maxillary first and second molars under 3% isoflurane anesthesia‡, using a #5 dental explorer for elevation. Four weeks after socket healing, custom screw‐shaped implants§ (6AL4V titanium, 1.0 mm length, 0.5 mm diameter) were placed at the tissue level in the first maxillary molar area. The implants were allowed to osseointegrate for 4 weeks^17^. Post tooth extraction and implant placement, mice received subcutaneous analgesics‖ (5 mg/kg, every 24 h for 3 days) and oral antibiotics¶ (850 µg/170 µg per mL) in their drinking water for 4 weeks. Osseointegration was assessed under anesthesia using buccolingual pressures on the implant head (Figure 1).

### 2.3 BP administration, peri‐implantitis induction, and euthanasia

Mice (12 per group) were randomly assigned to receive saline (vehicle) or 200 µg/kg zoledronic acid (ZA)# via intraperitoneal injections twice a week for 2 weeks. Weekly weight checks ensured consistent dosage.^18^, ^19^ After 2 weeks, ligatures** were placed around the implant heads on the left side. Control group implants did not undergo ligature placement.^17^ Intraperitoneal ZA administration continued for 6 weeks. Two groups were defined: vehicle (Veh) and ZA, each with healthy implant control (C) and ligature (L) sites. At study conclusion, animals were euthanized with isoflurane overdose, and their maxillae were photographed using a digital microscope.†† Maxillae were fixed in 10% formalin for 48 h, then preserved in 70% ethanol.

### 2.4 Micro‐computed tomography analysis (micro‐CT)

Maxillae were scanned using micro tomograph‡‡ (10 µm resolution) following Pirih et al. (2015).^17^ Linear bone height was measured with medical software§§, while volumetric analyses used micro‐CT analysis software.‖‖ Bone loss was measured from the implant head to the alveolar bone crest (ABC) in mesial and distal aspects. Volumetric bone loss was assessed following Wong et al. 2018.^20^

### 2.5 Histological assessment

Maxillae were decalcified in 15% ethylenediaminetetraacetic acid (EDTA) for 4 weeks. Ligatures were removed, and implants were unscrewed. Coronal cuts were made, and 5 µm sections were stained with hematoxylin and eosin (H&E). Digital imaging was done using digital pathology software.¶¶ Osteonecrosis was evaluated through empty lacunae counts.^18^ Statistical analysis was conducted for all samples (*n* = 12/group).

### 2.6 Osteoclastic activity

Tartrate‐resistant acid phosphatase (TRAP) assays assessed osteoclasts (*n* = 6/group). Osteoclasts with at least 3 nuclei in contact with the bone along the alveolar crest were counted and analyzed^21^. The analyses were conducted by a single‐blinded examiner (S.M.) who had undergone intra‐examiner calibration training (intraclass correlation coefficient [ICC] = 0.98).

### 2.7 Collagen assessment

Picrosirius red staining was used to assess collagen organization in bone. Type I collagen (yellow staining) plays a key role in bone matrix strength, while Type III collagen (green staining) is linked to vascularization.^22^ Observational analyses were performed (*n* = 3/group) (Figure 5B).

### 2.8 Immunohistochemical analysis

Neutrophil## and monocyte/macrophage (CD11b)*** expression was assessed on coronal sections (*n* = 6/group). Secondary antibodies included anti‐rabbit IgG††† and anti‐rat IgG.‡‡‡ ^23‐25^ Image analysis was done using an advanced image analysis software§§§ designed for digital pathology and biomarker research.

### 2.9 Statistical analysis

For linear and volumetric analysis, inflammatory infiltrate, osteoclast activity, bone necrosis assessment, and immunohistochemical (IHC) expressions, the data were normalized and presented as mean ± SEM. Two‐way analysis of variance (ANOVA) followed by Tukey's test was used (GraphPad, La Jolla, California, USA). Significance levels were *p≤0.05, ^†^ *p*≤0.01, ^‡^ *p* < 0.001, and ^§^ *p* < 0.0001.

## 3 RESULTS

### 3.1 Ligature increases peri‐implant soft tissue

Clinical evaluation of soft tissues revealed that the gingival tissue in the Veh‐C group appeared clinically normal, with no signs of tissue swelling. Similar findings were observed in the ZA‐C group. Ligature placement in both the Veh‐ and the ZA‐treated groups resulted in soft tissue edema. Qualitatively, there were no differences in the degree of soft tissue edema in the ZA‐L group compared to the Veh‐L groups (Figure 3A).

### 3.2 Ligature placement does not induce peri‐implant bone loss in the presence of ZA

**FIGURE 2:**
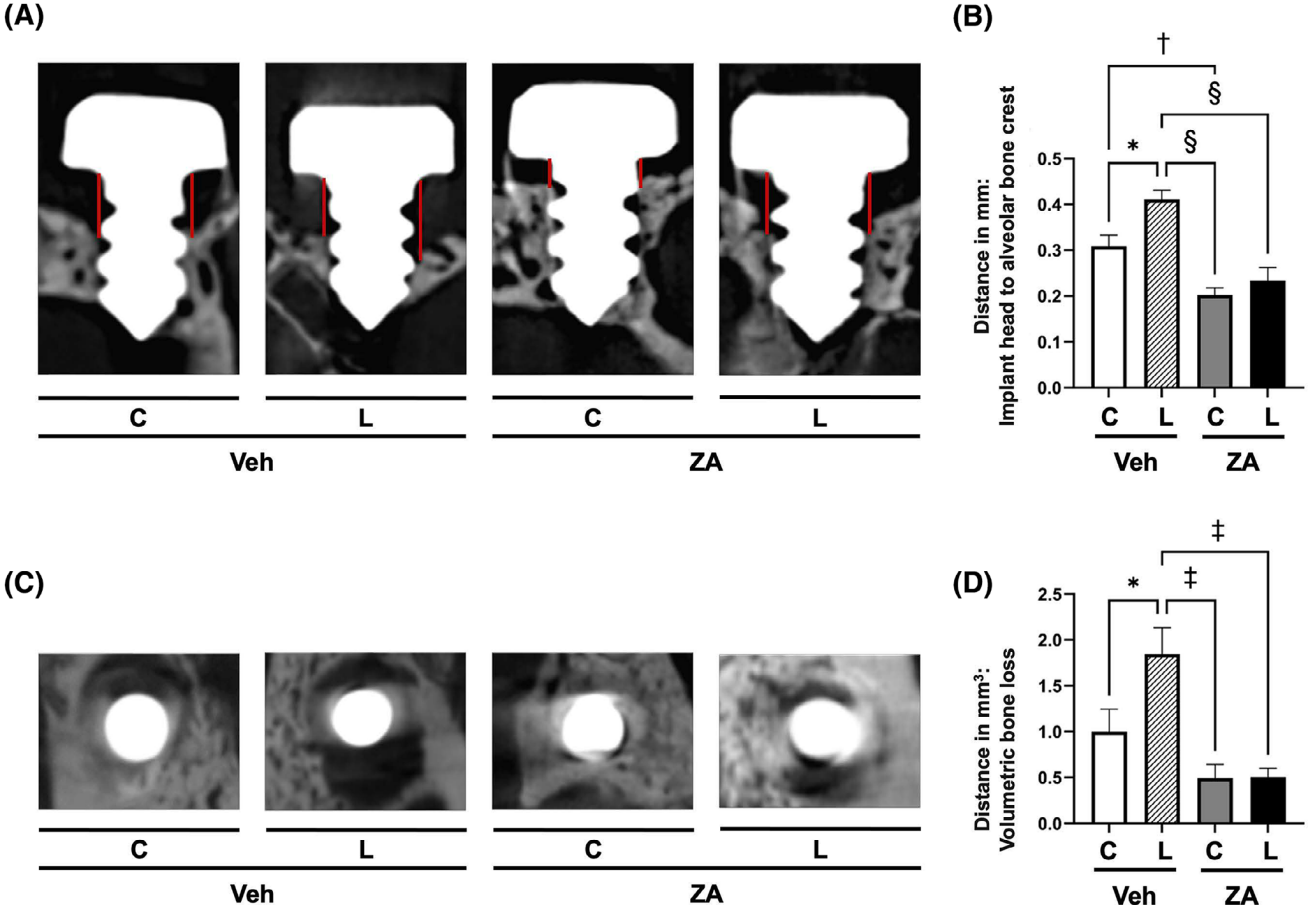
Micro‐CT assessment of hard tissues. (A) Representative reconstructions of the sagittal sections for all groups, 6 weeks after ligature placement. (B) Graph represents the average distance from the implant head to alveolar bone crest. (C) Representative reconstructions of the axial sections for all groups. (D) Graph represents the average circumferential volumetric bone loss. Data are mean ± standard error of the mean. *p≤0.05, ^†^ *p*≤0.01, ^‡^ *p* < 0.001, ^§^ *p* < 0.0001. One‐way ANOVA followed by a Tukey's test (*n* = 12 samples/group).

To evaluate linear and volumetric bone loss (*n* = 12 samples/group), we utilized micro‐CT.^26^ The Veh‐L group exhibited a statistically significant increase in bone loss compared to the Veh‐C group (*p* ≤ 0.05). No statistically significant difference in bone levels was observed between the ZA‐L and the ZA‐C groups. Moreover, the Veh‐L group demonstrated statistically significantly greater bone loss compared to the ZA‐L group (*p* < 0.0001) (Figure 2A and 2B).

The volumetric assessment paralleled the linear analysis (Figure 2C and 2D). The Veh‐L group exhibited a statistically significant increase in bone loss compared to the Veh‐C group (*p* ≤ 0.05). No significant difference in volumetric bone levels was observed between the ZA‐L and the ZA‐C groups. Moreover, the Veh‐L group displayed significantly greater bone loss compared to the ZA‐L group (*p* < 0.0001).

### 3.3 Ligature increases peri‐implant soft tissue inflammation

**FIGURE 3:**
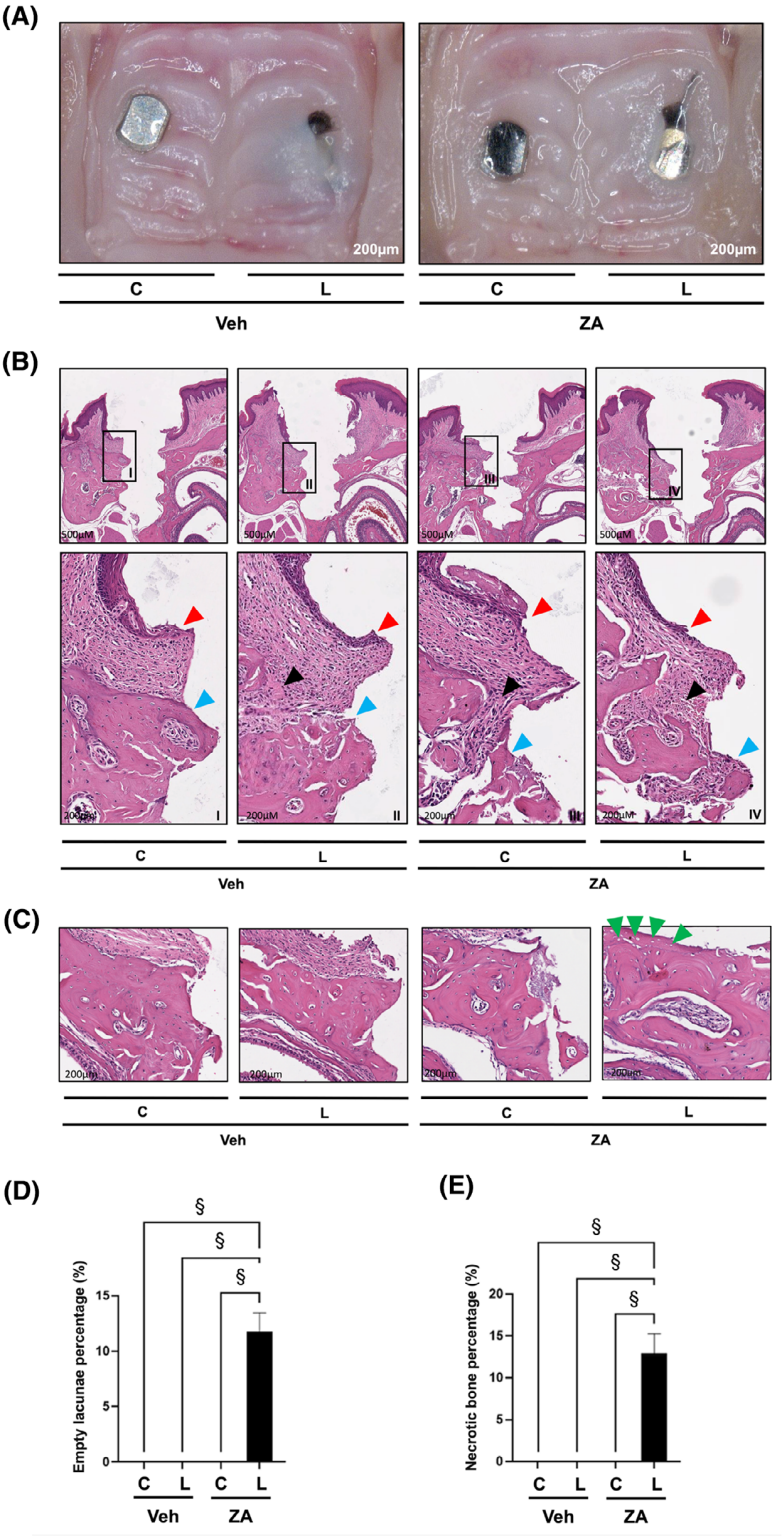
Clinical and histologic findings. (A) Clinical evaluation of soft tissues. Representative clinical images were taken from an occlusal view of the Veh and ZA groups, including their C and L sites, 6 weeks after ligature placement. (B) Representative coronal sections of H&E for all groups in the implant region, 6 weeks after ligature placement. Red arrows: marginal gingival epithelium. Black arrows: inflammatory infiltrate. Blue arrows: alveolar crest. Original magnification: 500 and 200 µm. (C) Empty lacunae and necrotic bone assessment. Representative coronal sections of H&E for all groups in the implant region. Green arrows: empty lacunae. Original magnification: 200 µm. (D) Graph represents the percentage of empty lacunae. (E) Graph represents the percentage of necrotic bone. Data are mean ± standard error of the mean. ^§^ *p* < 0.0001. One‐way ANOVA followed by a Tukey's test (*n* = 8 samples/group).

To qualitatively evaluate histomorphological and inflammatory changes, H&E staining was performed. In both Veh and ZA groups, the ligature sites showed exacerbated gingival inflammation, and alveolar bone resorption, compared to the control sites. Moreover, PMN (polymorphonuclear leukocytes) infiltration was increased on the ligature sites. Qualitatively comparison of PMN density between the Veh‐L and ZA‐L groups did not reveal differences (Figure 3B).

### 3.4 Empty lacunae and necrotic bone area are increased at the peri‐implant ligature sites with ZA

To assess necrotic bone, number of empty osteocytic lacunae were evaluated in H&E‐stained sections (Figure 3C). Empty lacunae were evident exclusively in the ZA‐L group. The percentage of necrotic bone, quantified by the area of bone composed of the empty osteocyte lacunae was also quantified. Necrotic bone areas were only observed in the ZA‐L group. Statistical analysis confirmed a statistically significant increase in empty lacunae in the ZA‐L group compared to all other groups (*p* < 0.001) (Figure 3D and 3E), confirming the presence of osteonecrosis in the ZA‐L group.

### 3.5 ZA decreases the number of osteoclasts at the peri‐implant ligature sites

**FIGURE 4:**
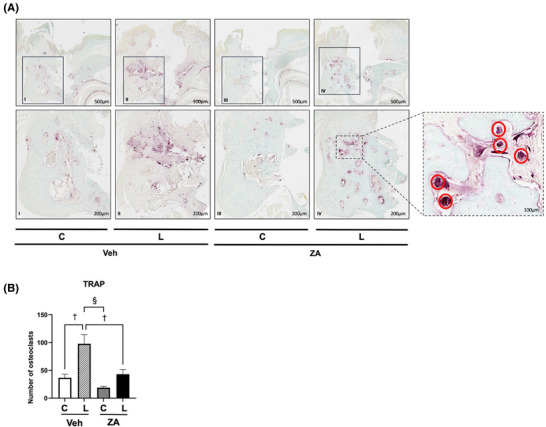
Osteoclasts assessment. (A) Representative coronal TRAP staining images of all groups in the implant region. Original magnification: 500 and 200 µm. Red circles indicate round osteoclasts detached from the bone due to apoptosis. Original magnification: 100 µm. (B) Graph represents the average and normalized osteoclast count in bone perimeter. Data are mean ± standard error of the mean. ^†^ *p*≤0.01, ^§^ *p* < 0.0001. One‐way ANOVA followed by a Tukey's test (*n* = 3 for all groups).

To assess osteoclast numbers, samples were stained with TRAP (*n* = 6 mice per group). Osteoclasts exhibiting 3 nuclei in contact with the bone in the buccal and palatal implant areas were counted and statistically analyzed. The Veh‐C group showed an increase in TRAP+ cells compared to the ZA‐C group (Figure 4A and 4B). The Veh‐L group exhibited a statistically significant higher number of osteoclasts compared to the Veh‐C group (*p* ≤ 0.01). No statistical difference was observed between the ZA‐L and the ZA‐C groups. Additionally, the Veh‐L group had a significantly higher number of osteoclasts compared to the ZA‐L group (*p* ≤ 0.01). Interestingly, only the ZA‐L group showed round osteoclasts detached from the bone as a consequence of apoptosis (Figure 4A).

### 3.6 Collagen is detached from the necrotic bone at the peri‐implant ligature sites in the presence of ZA

**FIGURE 5:**
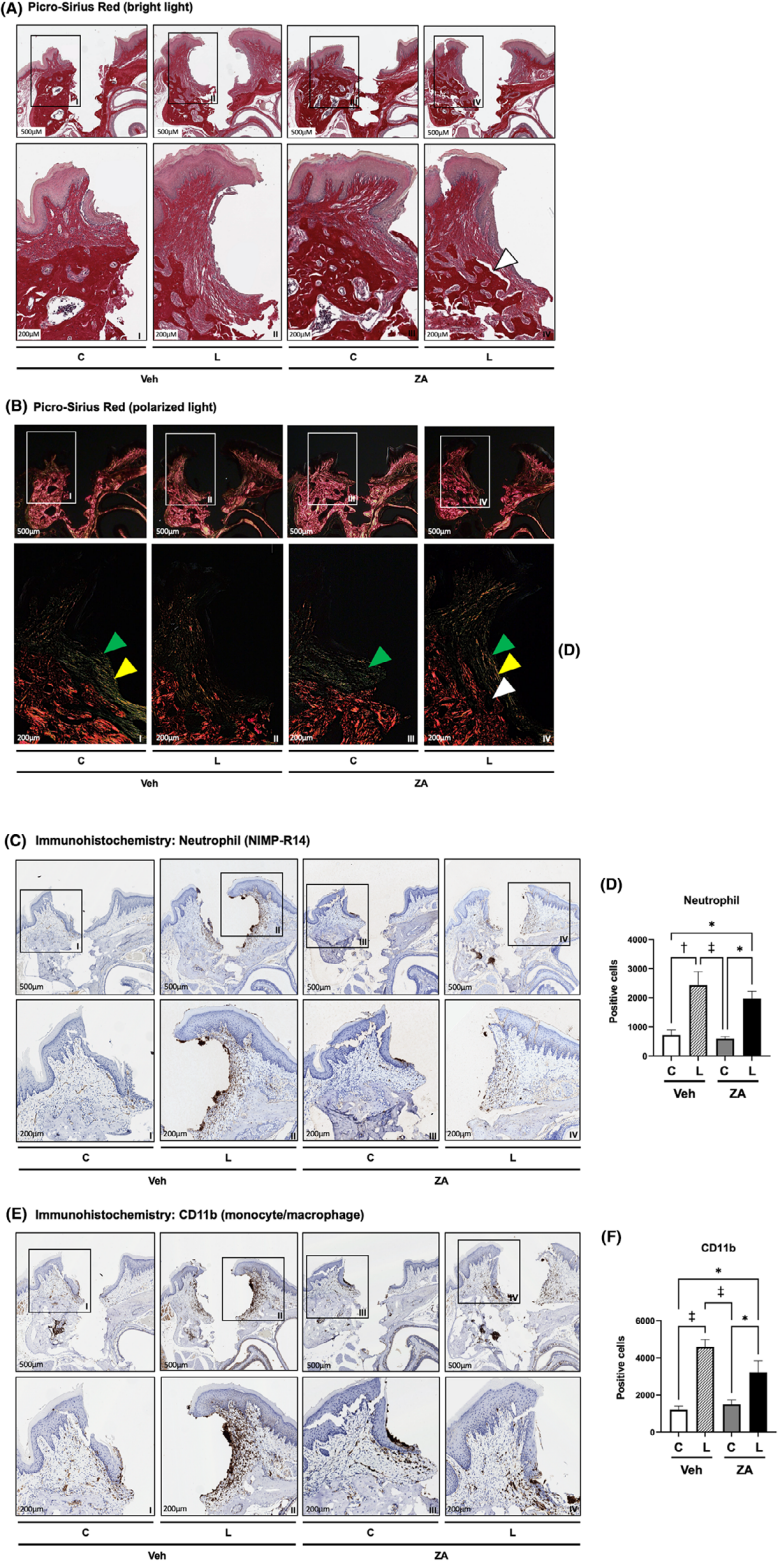
Collagen assessment. Representative coronal Picrosirius staining under (A) bright and (B) polarized light of all groups in the implant region, 6 weeks after ligature placement. Yellow arrows: Type I collagen. Green arrows: Type III collagen. White arrow: absence of epithelium attachment. Original magnification: 500 and 200 µm (*n* = 3 samples/group). Immunohistochemical assessment: Representative coronal immunohistochemistry for (C) neutrophil, and (E) CD11b. Original magnification: 500 and 200 µm. Graphs represent the average and normalized positive cells counting for (D) NIMP‐R14, and (F) monocyte/macrophage. Data are mean ± standard error of the mean. *p ≤ 0.05, ^†^ *p* ≤ 0.01, ^‡^ *p* < 0.001. One‐way ANOVA followed by a Tukey's test (*n* ≤ 9 samples/group).

To evaluate collagen organization, Picrosirius red staining was performed in tissue sections (*n* = 3 samples/group). Type I collagen, shown in yellow in Figure 5, contributes to the bone matrix structure. Type III collagen, shown in green in Figure 5, is associated with blood vessel formation and, consequently, vascularization. Under both bright light (Figure 5A) and polarized light (Figure 5B), a balance between collagen Type I (yellow) and Type III (green) was observed in the Veh‐C group. The ZA‐C group exhibited disorganized fibers and a reduction in Type I collagen fibers (yellow). In the Veh‐L group, the collagen displayed less symmetry, with disorganized fiber structure. Although, the ZA‐L group showed collagen Type I and Type III, soft tissue separation was evident from the bone (Figure 5B).

### 3.7 Ligature increases the number of neutrophils and monocyte/macrophages in the peri‐implant soft tissue

To evaluate neutrophil (NIMP‐R14) and monocyte/macrophage expression (CD11b), IHC was performed. The ligature groups demonstrated a statistically significant increase in neutrophils (NIMP‐R14+cells) compared to the Veh groups (Figure 5C and 5D) (*p* ≤ 0.01 and (*p* ≤ 0.05, respectively). Similarly, for monocyte/macrophages (CD11b+ cells), immunohistochemical analysis revealed an increased number of positive cells similar results, in both ligature‐treated groups compared to the respective control groups (Figure 5E and 5F).

## 4 DISCUSSION

Dental implants are a significant concern and are generally not recommended for patients receiving high‐dose BPs for the management of malignancy, due to the increased risk of MRONJ.^27^, ^28^ However, healthy individuals with stable dental implants may develop systemic conditions requiring BP treatment.^29^, ^30^ Therefore, our study aimed to evaluate whether inflammation around an osseointegrated dental implant could lead to MRONJ in mice treated with ZA. To address this question, this study utilized a peri‐implantitis murine model combined with oncologic dosages of ZA to elucidate the pathophysiological mechanisms underlying MRONJ at the cellular and tissue levels. This is the first study to simultaneously examine peri‐implant inflammation and MRONJ in a split‐mouth design in mice.

Clinically, the Veh‐L and ZA‐L groups exhibited peri‐implant tissue edema compared to their respective control groups. But no qualitative difference was observed between the Veh‐L and the ZA‐L groups. This corroborates with the current literature that ligature induces soft tissue edema,^31^, ^32^, ^33^ and also agrees with a study published in rats where a ligature induced similar amounts of inflammation in the Veh and ZA groups.^14^

Regarding the linear and volumetric bone loss, the micro‐CT analysis in our study revealed a clear and exciting difference between groups. Peri‐implant soft tissue inflammation was increased in the Veh‐L^31^, ^32^, ^33^, ^34^ and in the ZA‐L groups. However, when ZA was administered, there was no significant bone loss comparing the ZA‐Veh and ZA‐L groups. Endorsing this finding, a recent meta‐analysis showed that, in general, the use of BP for treating osteoporosis does not adversely affect implant therapy in patients. The assessment encompassed several factors, including peri‐implant marginal bone loss, MRONJ, and peri‐implantitis presence.^29^ Another animal model experiment evaluating socket healing found similar radiographic results, in which there was a similar bone appearance around the second molar socket of Veh or ZA animals.^19^

Histological analysis revealed information about soft tissue inflammation and PMN infiltration. The comparable PMN density between the Veh‐L and ZA‐L groups suggests a similar soft tissue inflammatory response. A closer examination of histomorphological and immunohistochemical changes enriches our understanding of the cellular processes of tissue inflammation. Our analysis also showed neutrophil and monocyte/macrophage staining exhibited statistically higher expression in the ligature groups compared to the control groups. Given that neutrophils serve as the first line of defense in the presence of inflammation, this endorses our findings.

When investigating the osteoclastic activity, our findings underscored the critical role of these bone‐resorbing cells in the pathogenesis of osteonecrosis. Osteoclasts, which are multinucleated cells responsible for bone resorption, play a pivotal role in maintaining bone homeostasis.^35^, ^36^ In the present study, groups treated with BPs exhibited lower osteoclast counts due to the drug's mechanism of action, which specifically inhibits osteoclast activity and promotes apoptosis of these cells.^37^ However, it is important to note that prolonged BP use may lead to an increase in the number and size of osteoclasts, as observed by^38^. Their study demonstrated that long‐term BP therapy can result in the formation of giant osteoclasts. Despite these larger and more numerous osteoclasts, their function remains impaired, with diminished resorptive capacity. This paradoxical effect highlights the complexity of BP action on bone remodeling, where osteoclast morphology and activity can be altered without restoring their bone‐resorptive function.

In the context of ligature‐induced inflammation, the inflammatory signals typically stimulate osteoclastogenesis and increase osteoclast activity to respond to bone loss. However, the presence of BPs counteracts this effect by suppressing osteoclast function and survival.^19^, ^39^ As a result, even in the inflammatory environment created by ligature placement, the BPs effectively limit the activity of osteoclasts, observed by the lower osteoclast counts compared to groups without BPs. This creates a complex scenario where, while BPs reduce osteoclast numbers and prevent further bone loss, they may also lead to an imbalance in bone remodeling that could contribute to conditions like osteonecrosis.^40^, ^41^, ^42^

In our study, only the ZA‐L group exhibited significant necrotic areas. These findings align with previous studies that have documented the occurrence of osteonecrosis in association with BP treatment regimens. For instance, research by Ruggiero et al.^2^ noted a prevalence of necrotic bone in patients receiving BP therapy, indicating a potential common mechanism of action that compromises bone integrity. Additionally, a study by de Molon et al.^43^ demonstrated that significant increases in empty osteocytic lacunae and osteonecrotic area were present in the diseased sites of mice on antiresorptive treatment. The significant differences in necrotic bone area observed in our study further corroborate these findings, highlighting a need for awareness regarding the risk of osteonecrosis in similar therapeutic contexts.

Picrosirius staining for collagen types I and III in our study provided a detailed view of collagen predominance and organization. Differences were observed, showing a separation of collagen fibers from the necrotic bone in the ZA‐L. Similarly, a recent murine study observed a lack of collagen fiber insertion in the necrotic bone of rats under ZA medication.^19^ While BPs primarily target bone tissue, they can also have effects on oral tissues, including inhibition of cell migration in gingival fibroblasts.^44^

Our murine model offers valuable translational insights into the pathophysiology of MRONJ, which could serve as a foundation for testing novel preventive strategies and therapeutic interventions. According to the American Association of Oral and Maxillofacial Surgeons (AAOMS), bone remodeling is critical in preventing and resolving MRONJ. This is supported by animal studies that evaluate the effects of BP withdrawal or the use of denosumab, highlighting their potential to mitigate the risks associated with MRONJ^2^.

While we acknowledge the limitations of our study, such as the sample size, female samples absence, and the acute nature of peri‐implantitis, it is essential to note that these limitations can serve as a roadmap for future research. Including female mice in future studies could provide insights into potential sex‐based differences in peri‐implant inflammation and bone remodeling, which may influence hormonal and immune system variations. Additionally, incorporating chronic inflammation models would more closely replicate the natural disease progression observed in clinical settings, enabling a better understanding of the long‐term effects of peri‐implantitis and BP treatment. Addressing these limitations in future research will enhance statistical rigor, allow a more comprehensive exploration of chronic inflammatory processes, and contribute to refining study designs for more robust conclusions.

## 5 CONCLUSION

This exciting study provides a novel model of MRONJ associated with peri‐implantitis in mice and sheds light on the multifaceted nature of MRONJ, revealing previously unexplored clinical, histological changes and intricate bone structural changes. With limited research addressing BPs and peri‐implant inflammation at an experimental level, our findings provide a strong foundation for future investigations. This work has the potential to pave the way for innovative prevention and treatment strategies for both conditions.

## AUTHOR CONTRIBUTIONS

Ana Bujila and Davi N. A. Silva are co‐first authors. Both contributed equally to the work, including conception and design, data acquisition, analysis, and interpretation, draft and critical review of the manuscript; Sepehr Monajemzadeh, Taciane M. da Silveira, Naseim Elzakra, Maísa Casarin, Kimberly Flores, and Clara Magyar contributed to analysis and critically revised the manuscript; Julie Marchesan, Reuben Kim, and Sotirios Tetradis contributed to conception and design, and critically revised the manuscript; Flavia Q. Pirih contributed to conception and design, contributed to analysis, interpretation, and critically revised the manuscript. All authors gave final approval and agreed to be accountable for all aspects of the work.

## CONFLICT OF INTEREST STATEMENT

The authors have nothing to disclose.

## Data Availability

The data that support the findings of this study are available from the corresponding author upon reasonable request.

## Footnotes

* The Jackson Laboratories (Bar Harbor, ME, USA).

† Bio Serve (Frenchtown, NJ, USA).

‡ Vet One (Boise, ID, USA).

§ D. P. Machining Inc. (La Verne, CA, USA).

‖ Carprofen – Zoetis (San Diego, CA, USA).

¶ Sulfamethoxazole/Trimethoprim – Zoetis (San Diego, CA, USA).

# Zoledronic Acid – NorthStar (Memphis, TN, USA).

** Fisher Scientific (Hampton, NH, USA).

†† Keyence (Osaka, Japan).

‡‡ SkyScan 1172 – SkyScan (Kontich, Belgium).

§§ Dolphin Software – Navantis (Toronto, Ontario, Canada).

‖‖ CTAn software (V.1.16 Bruker) – Bruker Micro‐CT (Kontich, Antwerp, Belgium).

¶¶ Aperio ImageScope (V11.1.2.752) – Leica Biosystems (Vista, CA, USA).

## Neutrophil (NIMP‐R14, Cat #AB2557, 1:150) – Abcam (Cambridge, England, United Kingdom).

*** Monocyte/macrophage (CD11b, Cat #AB133357, 1:150) – Abcam (Cambridge, England, United Kingdom).

††† Anti‐rabbit IgG (Cat #AB205718, 1:200) – Abcam, (Cambridge, England, United Kingdom).

‡‡‡ Anti‐rat IgG (Cat #AB205720, 1:200) – Abcam, (Cambridge, England, United Kingdom).

§§§ Tissue Studio v. 4.3.1 – Definiens AG (Munich, Bavaria, Germany).

## References

[1] Mbese Z , Aderibigbe BA . Bisphosphonate‐based conjugates and derivatives as potential therapeutic agents in osteoporosis, bone cancer and metastatic bone cancer. Int J Mol Sci. 2021;22(13). doi:10.3390/ijms22136869 PMC8268474 34206757

[2] Ruggiero SL , Dodson TB , Aghaloo T , Carlson ER , Ward BB , Kademani D . American association of oral and maxillofacial surgeons' position paper on medication‐related osteonecrosis of the jaws‐2022 update. J Oral Maxillofac Surg. 2022;80(5):920‐943. doi:10.1016/j.joms.2022.02.008 35300956

[3] Lo JC , O'Ryan FS , Gordon NP , et al. Prevalence of osteonecrosis of the jaw in patients with oral bisphosphonate exposure. J Oral Maxillofac Surg. 2010;68(2):243‐253. doi:10.1016/j.joms.2009.03.050 19772941 PMC10159647

[4] Khan AA , Rios LP , Sandor GK , et al. Bisphosphonate‐associated osteonecrosis of the jaw in Ontario: a survey of oral and maxillofacial surgeons. J Rheumatol. 2011;38(7):1396‐1402. doi:10.3899/jrheum.100221 21498483

[5] Pazianas M , Miller P , Blumentals WA , Bernal M , Kothawala P . A review of the literature on osteonecrosis of the jaw in patients with osteoporosis treated with oral bisphosphonates: prevalence, risk factors, and clinical characteristics. Clin Ther. 2007;29(8):1548‐1558. doi:10.1016/j.clinthera.2007.08.008 17919538

[6] Ehrenstein V , Heide‐Jorgensen U , Schiodt M , et al. Osteonecrosis of the jaw among patients with cancer treated with denosumab or zoledronic acid: results of a regulator‐mandated cohort postauthorization safety study in Denmark, Norway, and Sweden. Cancer. 2021;127(21):4050‐4058. doi:10.1002/cncr.33802 34310704

[7] Saad F , Brown JE , Van Poznak C , et al. Incidence, risk factors, and outcomes of osteonecrosis of the jaw: integrated analysis from three blinded active‐controlled phase III trials in cancer patients with bone metastases. Ann Oncol. 2012;23(5):1341‐1347. doi:10.1093/annonc/mdr435 21986094

[8] Sher J , Kirkham‐Ali K , Luo JD , Miller C , Sharma D . Dental implant placement in patients with a history of medications related to osteonecrosis of the jaws: a systematic review. J Oral Implantol. 2021;47(3):249‐268. doi:10.1563/aaid-joi-D-19-00351 32699903

[9] Ting M , Huynh BH , Woldu HG , Gamal I , Suzuki JB . Clinical impact on dental implant survival in patients taking antiresorptive medications: a systematic review and meta‐analysis. J Oral Implantol. 2023;49(6):599‐615. doi:10.1563/aaid-joi-D-21-00160 37905745

[10] Limones A , Saez‐Alcaide LM , Diaz‐Parreno SA , Helm A , Bornstein MM , Molinero‐Mourelle P . Medication‐related osteonecrosis of the jaws (MRONJ) in cancer patients treated with denosumab VS. zoledronic acid: a systematic review and meta‐analysis. Med Oral Patol Oral Cir Bucal. 2020;25(3):e326‐e336. doi:10.4317/medoral.23324 32271321 PMC7211372

[11] Schwarz F , Derks J , Monje A , Wang HL . Peri‐implantitis. J Clin Periodontol. 2018;45(20):S246‐S266. Suppl. doi:10.1111/jcpe.12954 29926484

[12] Seki K , Namaki S , Kamimoto A , Hagiwara Y . Medication‐related osteonecrosis of the jaw subsequent to peri‐implantitis: a case report and literature review. J Oral Implantol. 2021;47(6):502‐510. doi:10.1563/aaid-joi-D-19-00385 33270837

[13] Tempesta A , Capodiferro S , Mauceri R , et al. Peri‐implantitis‐like medication‐related osteonecrosis of the jaw: clinical considerations and histological evaluation with confocal laser scanning microscope. Oral Dis. 2022;28(6):1603‐1609. doi:10.1111/odi.13873 33844402 PMC9541517

[14] Quintao Manhanini Souza E , Felipe Toro L , Franzao Ganzaroli V , et al. Peri‐implantitis increases the risk of medication‐related osteonecrosis of the jaws associated with osseointegrated implants in rats treated with zoledronate. Scientific Reports 2024:627. doi:10.1038/s41598-023-49647-4 38182598 PMC10770413

[15] Kwon TG , Lee CO , Park JW , Choi SY , Rijal G , Shin HI . Osteonecrosis associated with dental implants in patients undergoing bisphosphonate treatment. Clin Oral Implants Res. 2014;25(5):632‐640. doi:10.1111/clr.12088 23278625

[16] Percie du Sert N , Hurst V , Ahluwalia A , et al. The ARRIVE guidelines 2.0: updated guidelines for reporting animal research. PLoS Biol. 2020;18(7):e3000410. doi:10.1371/journal.pbio.3000410 32663219 PMC7360023

[17] Pirih FQ , Hiyari S , Barroso AD , et al. Ligature‐induced peri‐implantitis in mice. J Periodontal Res. 2015;50(4):519‐524. doi:10.1111/jre.12234 25244403 PMC4368501

[18] Soundia A , Hadaya D , Esfandi N , et al. Osteonecrosis of the jaws (ONJ) in mice after extraction of teeth with periradicular disease. Bone. 2016;90:133‐141. doi:10.1016/j.bone.2016.06.011 27327410 PMC5471352

[19] Soundia A , Hadaya D , Esfandi N , et al. Zoledronate impairs socket healing after extraction of teeth with experimental periodontitis. J Dent Res. 2018;97(3):312‐320. doi:10.1177/0022034517732770 28954199 PMC5833182

[20] Wong RL , Hiyari S , Yaghsezian A , et al. Early intervention of peri‐implantitis and periodontitis using a mouse model. J Periodontol. 2018;89(6):669‐679. doi:10.1002/JPER.17-0541 29520950 PMC8607848

[21] Chaichanasakul T , Kang B , Bezouglaia O , Aghaloo TL , Tetradis S , Diverse osteoclastogenesis of bone marrow from mandible versus long bone. J Periodontol. 2014;85(6):829‐836. doi:10.1902/jop.2013.130376 24003963 PMC4472369

[22] Montes GS , Junqueira LC . The use of the Picrosirius‐polarization method for the study of the biopathology of collagen. Mem Inst Oswaldo Cruz. 1991;86(3):1‐11. doi:10.1590/s0074-02761991000700002 1726969

[23] Guang M , Huang B , Yao Y , Zhang L , Yang B , Gong P . Effects of vascular endothelial growth factor on osteoblasts around dental implants in vitro and in vivo. J Oral Sci. 2017;59(2):215‐223. doi:10.2334/josnusd.16-0406 28637981

[24] Hayashi H , Nakahama K , Sato T , et al. The role of Mac‐1 (CD11b/CD18) in osteoclast differentiation induced by receptor activator of nuclear factor‐kappaB ligand. FEBS Lett. 2008;582(21‐22):3243‐3248. doi:10.1016/j.febslet.2008.08.023 18775427

[25] Park‐Min KH , Lee EY , Moskowitz NK , et al. Negative regulation of osteoclast precursor differentiation by CD11b and beta2 integrin‐B‐cell lymphoma 6 signaling. J Bone Miner Res. 2013;28(1):135‐149. doi:10.1002/jbmr.1739 22893614 PMC3522783

[26] Kim Y , Brodt MD , Tang SY , Silva MJ . MicroCT for scanning and analysis of mouse bones. Methods Mol Biol. 2021;2230:169‐198. doi:10.1007/978-1-0716-1028-2_11 33197015 PMC8409170

[27] Owen B , Bradley H . Is it safe to place implants in patients at risk of MRONJ?. Evid Based Dent. 2021;22(3):108‐109. doi:10.1038/s41432-021-0196-9 34561663

[28] Andersen SWM , Ottesen C , Gotfredsen K , Jensen SS , Kofod T , Schiodt M . Outcome of healing after dental implant placement in patients with cancer on high‐dose antiresorptive medications: a prospective feasibility study. Oral Maxillofac Surg. 2023;27(1):89‐100. doi:10.1007/s10006-022-01042-5 35084584

[29] Stavropoulos A , Bertl K , Pietschmann P , Pandis N , Schiodt M , Klinge B , The effect of antiresorptive drugs on implant therapy: systematic review and meta‐analysis. Clin Oral Implants Res. 2018;29 Suppl 18:54‐92. doi:10.1111/clr.13282 30306695

[30] Walter C , Al‐Nawas B , Wolff T , Schiegnitz E , Grotz KA . Dental implants in patients treated with antiresorptive medication—a systematic literature review. Int J Implant Dent. 2016;2(1):9. doi:10.1186/s40729-016-0041-7 27747701 PMC5005701

[31] Hiyari S , Naghibi A , Wong R , et al. Susceptibility of different mouse strains to peri‐implantitis. J Periodontal Res. 2018;53(1):107‐116. doi:10.1111/jre.12493 29044525 PMC5760471

[32] Hiyari S , Wong RL , Yaghsezian A , et al. Ligature‐induced peri‐implantitis and periodontitis in mice. J Clin Periodontol. 2018;45(1):89‐99. doi:10.1111/jcpe.12817 28921659 PMC5774657

[33] Wong RL , Hiyari S , Yaghsezian A , et al. Comparing the healing potential of late‐stage periodontitis and peri‐implantitis. J Oral Implantol. 2017;43(6):437‐445. doi:10.1563/aaid-joi-D-17-00157 29064761

[34] Yuan S , Wei Y , Jiang W , et al. CCR2 is a potential therapeutic target in peri‐implantitis. J Clin Periodontol. 2024;51(3):354‐364. doi:10.1111/jcpe.13916 38111083

[35] Kim JM , Lin C , Stavre Z , Greenblatt MB , Shim JH . Osteoblast‐osteoclast communication and bone homeostasis. Cells. 2020;9(9). doi:10.3390/cells9092073 PMC7564526 32927921

[36] Omi M , Mishina Y . Roles of osteoclasts in alveolar bone remodeling. Genesis. 2022;60(8‐9):e23490. doi:10.1002/dvg.23490 35757898 PMC9786271

[37] Drake MT , Clarke BL , Khosla S , Bisphosphonates: mechanism of action and role in clinical practice. Mayo Clin Proc. 2008;83(9):1032‐1045. doi:10.4065/83.9.1032 18775204 PMC2667901

[38] Weinstein RS , Roberson PK , Manolagas SC . Giant osteoclast formation and long‐term oral bisphosphonate therapy. N Engl J Med. 2009;360(1):53‐62. doi:10.1056/NEJMoa0802633 19118304 PMC2866022

[39] Goes P , Melo IM , Dutra CS , Lima AP , Lima V , Effect of alendronate on bone‐specific alkaline phosphatase on periodontal bone loss in rats. Arch Oral Biol. 2012;57(11):1537‐1544. doi:10.1016/j.archoralbio.2012.07.007 23062673

[40] Aguirre JI , Castillo EJ , Kimmel DB. Preclinical models of medication‐related osteonecrosis of the jaw (MRONJ). Bone. 2021;153:116184. doi:10.1016/j.bone.2021.116184 34520898 PMC8743993

[41] Tamaoka J , Takaoka K , Hattori H , et al. Osteonecrosis of the jaws caused by bisphosphonate treatment and oxidative stress in mice. Exp Ther Med. 2019;17(2):1440‐1448. doi:10.3892/etm.2018.7076 30680026 PMC6327453

[42] Williams DW , Ho K , Lenon A , et al. Long‐term ligature‐induced periodontitis exacerbates development of bisphosphonate‐related osteonecrosis of the jaw in mice. J Bone Miner Res. 2022;37(7):1400‐1410. doi:10.1002/jbmr.4614 35598324 PMC9386631

[43] de Molon RS , Shimamoto H , Bezouglaia O , et al. OPG‐Fc but not zoledronic acid discontinuation reverses osteonecrosis of the jaws (onj) in mice. J Bone Miner Res. 2015;30(9):1627‐1640. doi:10.1002/jbmr.2490 25727550 PMC4995600

[44] Chang J , Hakam AE , McCauley LK . Current understanding of the pathophysiology of osteonecrosis of the jaw. Curr Osteoporos Rep. 2018;16(5):584‐595. doi:10.1007/s11914-018-0474-4 30155844

